# Modelling Analysis of a Membrane-Based Fresh Air Processing System

**DOI:** 10.3390/membranes12101022

**Published:** 2022-10-20

**Authors:** Sebastian Englart, Krzysztof Rajski

**Affiliations:** Faculty of Environmental Engineering, Wrocław University of Science and Technology, 50377 Wrocław, Poland

**Keywords:** dehumidification, evaporative cooling, liquid desiccant, heat and mass transfer, hollow-fiber membrane, numerical simulation, regeneration

## Abstract

The current standard of residential buildings is based on well-insulated and airtight construction as an effective way to reduce primary energy consumption. On the other hand, this intensifies the issue of the indoor air quality. In this paper, the applicability of a hybrid dehumidification/evaporative cooling system for the processing and supply of fresh air is evaluated. The proposed novel system employs cross-flow hollow-fiber membrane modules. To provide a better insight into this novel approach, the system’s performance is numerically investigated using a validated mathematical model. The proposed fresh air processing system provides supply air parameters in or close to the summer comfort range. The analyzed system is characterized by a high coefficient of performance (COP) of up to 33.2 at an outdoor air temperature in the range of 30 °C to 38 °C and a humidity ratio in the range of 8 g/kg to 14 g/kg. Moreover, a temperature difference of up to 9.7 °C can be obtained after the two-stage membrane processing and the mixing process.

## 1. Introduction

The increasing amount of time spent indoors is a growing concern in modern society. As the time of exposure to indoor pollutants is much longer than that of outdoor pollutants, indoor air quality plays an essential role in ensuring public health [1,2]. Moreover, the quality of the indoor environment affects the comfort and productivity of the occupants [3]. On the other hand, buildings account for about one-third of the global energy consumption and one-quarter of CO_2_ emissions [4]. Therefore, the energy-saving potential of this sector has been increased worldwide by the improvement of the airtightness of the building envelope [1,5]. It should be emphasized that residential buildings are a key area of concern in efforts to reduce primary energy consumption [5,6]. However, these well-insulated and airtight buildings have to overcome the intensified issue of indoor air quality. Hence, it is of great importance for researchers to solve the problem of indoor air pollution in residential buildings. An effective way to improve the indoor air quality is to supply outdoor air through the ventilation system. As a result, indoor pollutants can be diluted [7,8]. As the main ventilation methods, mechanical and natural ventilation can be distinguished. However, mechanical ventilation can be considered as a reliable and organized method for meeting indoor air quality requirements [7].

It should be mentioned that the supplied outdoor air has to be treated in terms of temperature and humidity in order to meet the thermal requirements for indoor air. As a result, additional energy consumption is necessary in order to supply sufficient fresh air to the room [1]. This creates room for improvements effected by reducing the building energy use through novel heating, ventilation, and air conditioning (HVAC) solutions instead of the commonly used vapor compression air conditioning systems [9].

Membrane-based HVAC systems can be considered a promising alternative to conventional systems. Hence, the membrane-based liquid desiccant air conditioning systems have been extensively investigated both experimentally and numerically due to the advantages of their high efficiency without liquid water condensation and capacity for regeneration using low-grade heat (solar energy) [9,10,11]. In addition, a hollow-fiber-type membrane improves the dehumidification performance due to the higher packing density [9,12]. Peng and Cao proposed a novel hybrid-connected two-stage liquid dehumidification fresh air system [13]. By combining two liquid desiccants (calcium chloride (CaCl_2_) and lithium chloride (LiCl)), the deep dehumidification of outdoor fresh air was achieved using low-temperature regeneration heat sources, a with higher energy efficiency ratio (EER) and exergy efficiency. Englart and Rajski [14] examined an outdoor air system that employs a cross-flow hollow-fiber membrane module. An improvement of 28–134% in the moisture removal rate was obtained in comparison to the conventional system. It was shown that the novel system offers the ability for proper outdoor air treatment in subtropical zones. Zhang et al. [15] applied hollow-fiber membranes to a cross-flow dehumidifier. The performance of the liquid desiccant dehumidification system was numerically investigated based on a mathematical model, which was validated against experimental results. The authors concluded that the proposed system, using potassium formate (KCOOH) as a desiccant, could be more beneficial compared with other systems using conventional liquid desiccants.

On the other hand, experimental and numerical studies of the cooling performance of hollow-fiber-membrane evaporative coolers were carried out. These systems can offer an effective strategy for eliminating the water droplet carryover issue and bacterial growth that affect the indoor air quality. The applicability of a hollow-fiber-membrane evaporative cooler was investigated by Li et al. [16]. The obtained outlet air temperature and relative humidity satisfied the thermal comfort requirements of hot and dry regions, such as Northwest China. Moreover, the possibility of using a membrane technique was investigated in [17], resulting in satisfactory energy efficiency ratio indicators. Cui et al. [18] performed in-depth numerical studies of the air treatment process considering the impacts of the inlet air conditions, the feed water velocity, and the geometric dimensions. The proposed membrane-based module achieved a wet bulb effectiveness of up to 0.73.

Moreover, hybrid dehumidification/evaporative cooling systems based on hollow-fiber modules have also been described. Jradi and Riffat [19] employed a hollow-fiber-based core for evaporative cooling and liquid desiccant dehumidification. They concluded that the proposed system provides a promising alternative for the maintenance of thermal comfort and humidity control. Abdollahi et al. [20] revealed the concept of a two-step membrane air conditioning system. It consists of a dehumidifier module and an evaporative cooling module connected in series. The thermal comfort of the occupants can be ensured with the use of the proposed system through the outdoor air treatment at temperatures up to 13.5 °C, with a humidity ratio of 12 g/kg.

Based on the conducted literature review, some gaps can be identified in the state of the art. To the best of our knowledge, no studies have been conducted in order to investigate the two-step membrane air conditioning system that combines a stage of dehumidification with a stage of evaporative cooling in parallel. Based on this explanation, the proposed fresh air processing system is novel in terms of its structural design and flow arrangement. Therefore, this approach can be identified as the main novelty of this study. As a further novelty, the comprehensive analysis is carried out using an experimentally validated model so as to evaluate the fresh air processing performance, where cross-flow hollow-fiber membrane modules are employed. The main objective of this study is to investigate the process of mixing the two separately processed outdoor airflows to provide sufficient fresh airflow to a conditioned room under the summer conditions of a temperate climate.

Combining all the above-mentioned issues together, here, an attempt is made to evaluate the applicability of a hybrid dehumidification/evaporative cooling system for the processing of fresh air as a unique alternative to the solutions available in the literature. This system has the following key features:

- An ability to provide fresh air for maintaining an acceptable indoor air quality in residential buildings.
- An ability to supply the pre-cooled and pre-dehumidified fresh air to meet the thermal requirements for indoor air.
- An ability to enabling the use of local air condition units based on circulated air alone to cover the internal cooling load.

## 2. Materials and Methods

**Figure 1:**
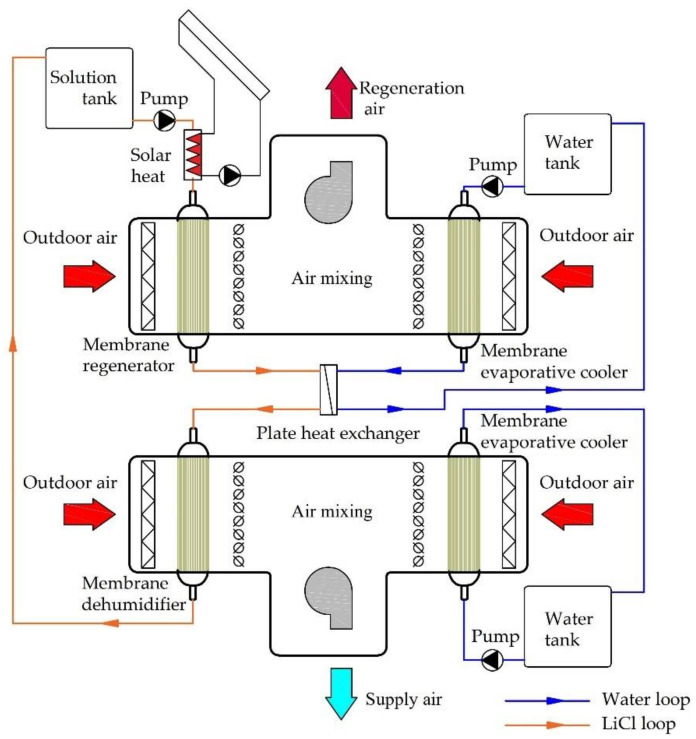
Membrane-based fresh air processing system.

The proposed system for obtaining outdoor air parameters corresponding to or close to indoor comfort parameters is shown in Figure 1. The system contains a system for outdoor air handling and a system for the regeneration of the liquid desiccant solution. The processing of the fresh air is based on air dehumidification, air evaporative cooling, and combination of two air streams. Dehumidification and evaporative cooling are carried out in the membrane modules. A 35% LiCl solution is used as the liquid desiccant. A membrane module is also used to regenerate the solution. It was assumed that, before regeneration, the solution heating takes place in a heat exchanger supplied with the heating medium by the solar collectors. Before the membrane dehumidifier is activated, the LiCl solution requires cooling. The cooling of the solution is achieved by a plate heat exchanger (PHE). The cooling water for the solution in the PHE is prepared in an additional membrane module.

**Figure 2:**
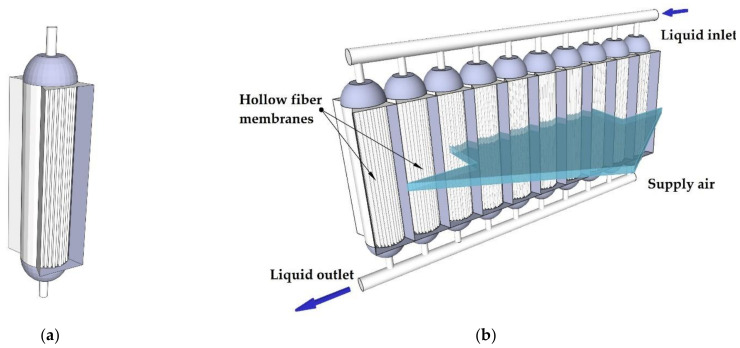
View of the membrane module for evaporative cooling or air dehumidification: (**a**) single hollow-fiber membrane module; (**b**) multimodule system.

In the presented system, polypropylene (PP) capillary membrane modules are used for air dehumidification, solution regeneration, air evaporative cooling, and water cooling. The structure of a single cross-flow membrane module is shown in Figure 2a. The parameters of a single module are summarized in Table 1. To increase the heat and mass transfer area, a single module was incorporated into a system of ten modules (Figure 2b).

### 2.1. Mathematical Modeling

#### 2.1.1. Membrane Module

**Figure 3:**
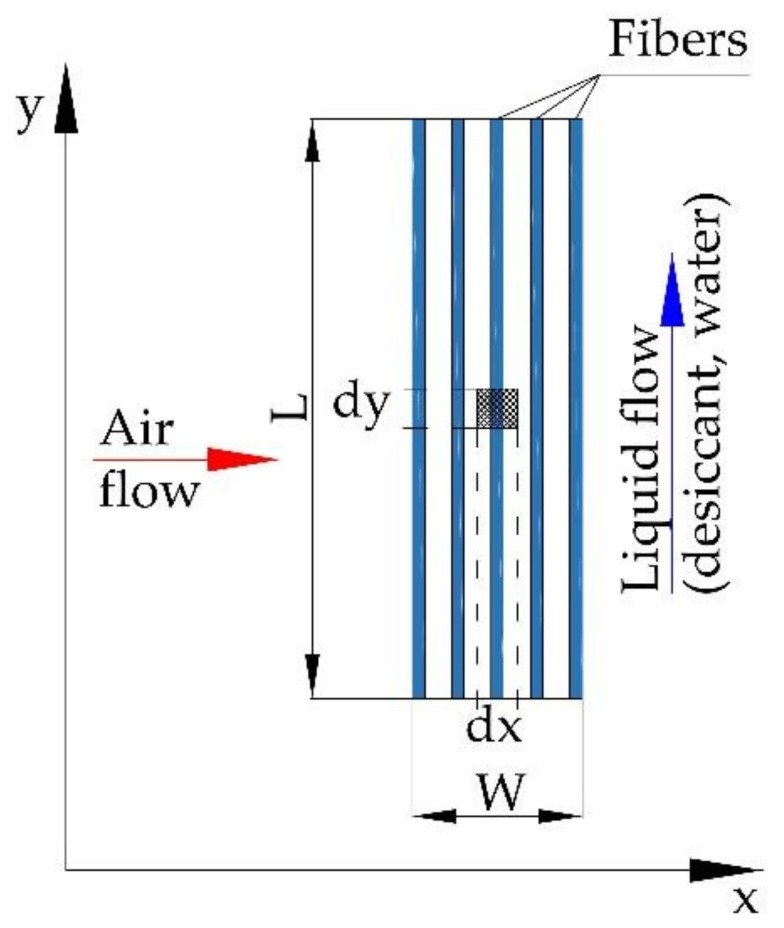
The coordinate system of the membrane module for the mathematical model.

A mathematical model based on the principles of the conservation of heat and mass was created to determine the air temperature and air humidity ratios and liquid parameters for a membrane evaporative cooler (MEC) and membrane dehumidifier (MD), or membrane regenerator (MR). A schematic view of the membrane module coordinate system is shown in Figure 3. The air stream flows along the *x*-axis, while the liquid (water or desiccant) flows along the *y*-axis.

The mathematical model is based on a series of basic assumptions:

- The modelling is considered in the midplane;
- The model is based on a two-dimensional cross-flow;
- The air and liquid flows are fully developed and laminar;
- The thermophysical properties of the fluids are constant;
- The amount of heat exchanged between the system and the surroundings is negligible;
- The axial heat and moisture transfer in the membrane are ignored;
- The changes in the air and liquid mass flow rates along the flow direction are negligible.

Using the above assumptions, a set of Equations (1)–(10) are derived, which are shown in Table 2 and Table 3.

The overall heat and mass transfer coefficients for a membrane module are determined as the sum of the individual resistances. Hence, the overall heat and mass transfer coefficients can be obtained using Equations (11) and (12), respectively:

(11) $$H_{ol}=\frac{1}{R_{hl}+R_{hm}+R_{ha}}$$

(12) $$K_{ol}=\frac{1}{R_{kl}+R_{km}+R_{ka}}$$

where:

(13) $$R_{hl}=\frac{1}{h_{l}}\left(\frac{d_{o}}{d_{i}}\right),R_{hm}=\frac{\delta }{\lambda_{m}}\left(\frac{d_{o}}{d_{\ln}}\right),R_{ha}=\frac{1}{h_{a}},R_{kl}=\frac{1}{k_{le}}\left(\frac{d_{o}}{d_{i}}\right),R_{km}=\frac{\delta }{D_{vm}}\left(\frac{d_{o}}{d_{\ln}}\right),R_{ka}=\frac{1}{k_{a}}$$

When using a non-salt solution, the resistance in the liquid phase can be considered negligible [24]. The physical properties of the solution were determined based on [23]. The equations for determining the various parameters used to calculate the resistance are summarized in Table 4.

The boundary conditions on the air side are:

(24) $$x=0,t_{a}=t_{a1},\omega_{a}=\omega_{a1}$$

The boundary conditions on the water side are:

(25) $$y=0,t_{w}=t_{w1},\omega_{w}=\omega_{w1}$$

The boundary conditions on the solution side are:

(26) $$y=0,t_{s}=t_{s1},\omega_{s}=\omega_{s1}$$

The water inlet temperature can be calculated using Equation (27):

(27) $$t_{w1}=t_{a}{}_{1}-\frac{h_{v}K_{ol}\rho_{a}\left(\omega_{w1}-\omega_{a}{}_{1}\right)}{H_{ol}}$$

In addition, the inlet solution temperature is equal to the outlet solution temperature from the PHE. The mathematical model was solved by employing a program written in the Mathcad environment, using a numerical procedure for solving ordinary differential equations with a given initial value.

#### 2.1.2. Solution Regeneration System

In the analyzed system, the LiCl solution requires heating before the regeneration and cooling prior to dehumidification. The heat and cooling balance of the solution side is summarized in Table 5.

Moreover, the analysis of the heat transfer between the solution and water in PHE can be performed based the ε-NTU method. The heat exchanger effectiveness can be expressed as follows [14]:

(36) $$\varepsilon =\frac{1-e^{-NTU\left(1-C_{r}\right)}}{1-C_{r}e^{-NTU\left(1-C_{r}\right)}}$$

(37) $$\varepsilon =\frac{{\dot{m}}_{{}_{w}}c_{pw}\left(t_{w2,reg}-t_{w1,reg}\right)}{{\dot{m}}_{{}_{s}}c_{ps}\left(t_{s2,reg}-t_{w1}{}_{,reg}\right)}$$

where:

(38) $$C_{r}=\frac{{\dot{m}}_{{}_{s}}c_{ps}}{{\dot{m}}_{{}_{w}}c_{pw}}$$

#### 2.1.3. Air Mixing

In a mixture of air streams, the end point of the mixture depends on the initial parameters of each mixed air stream, that is, the dehumidified air stream and the evaporatively cooled air stream. Hence, the air temperature and the air humidity ratio after mixing can be determined from the following equations:

(39) $$t_{a3}=\frac{{\dot{m}}_{{}_{a,EC}}t_{a2,EC}+{\dot{m}}_{{}_{a,D}}t_{a2,D}}{{\dot{m}}_{{}_{a,EC}}+{\dot{m}}_{{}_{a,D}}}$$

(40) $$\omega_{a3}=\frac{{\dot{m}}_{{}_{a,EC}}\omega_{a2,EC}+{\dot{m}}_{{}_{a,D}}\omega_{a2,D}}{{\dot{m}}_{{}_{a,EC}}+{\dot{m}}_{{}_{a,D}}}$$

### 2.2. Validation of the Mathematical Model

**Figure 4:**
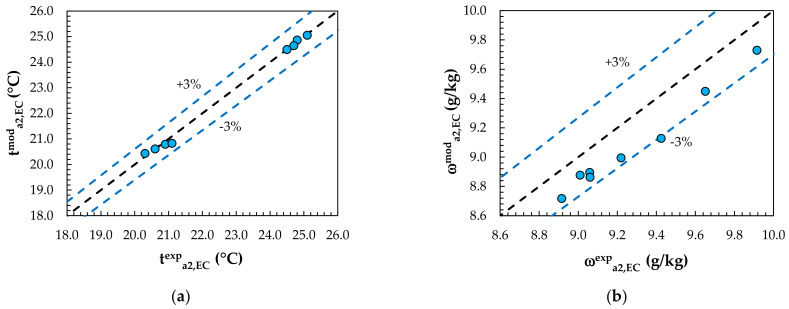
Comparison of model values with experimental results for evaporative cooling: (**a**) outlet air temperature; (**b**) outlet air humidity ratio.

**Figure 5:**
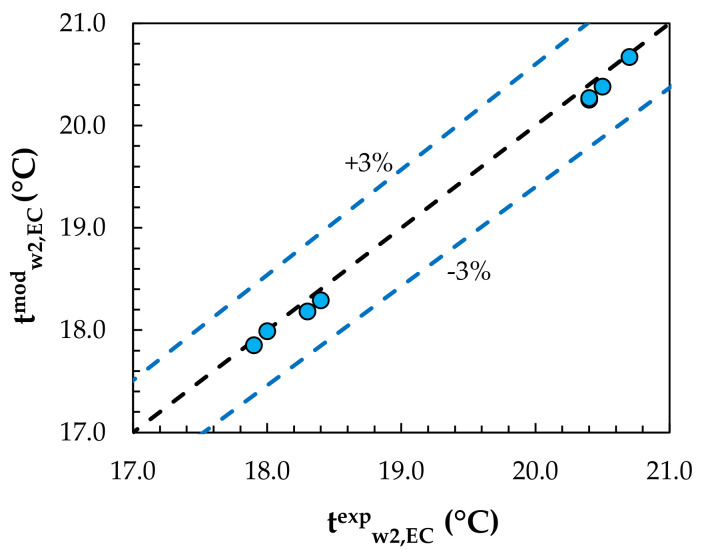
Comparison of model values with experimental results for the water temperature.

**Figure 6:**
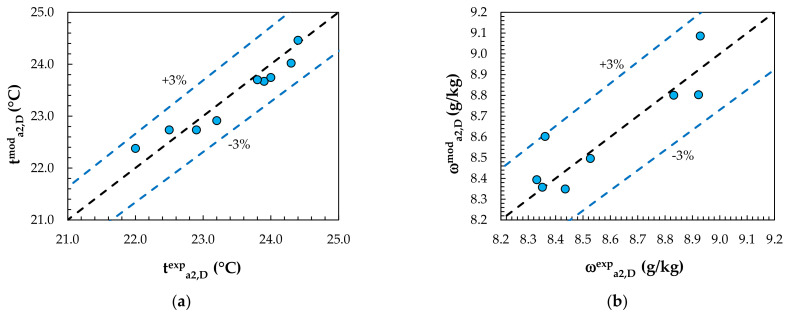
Comparison of model values with experimental results for dehumidification: (**a**) outlet air temperature; (**b**) outlet air humidity ratio.

**Figure 7:**
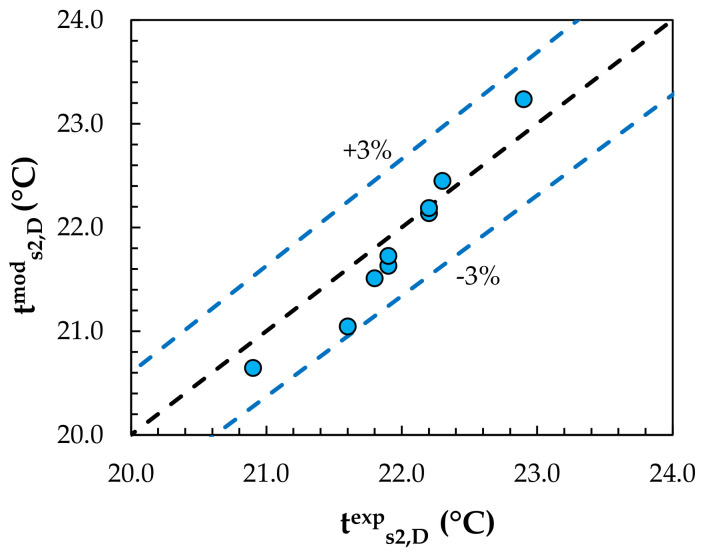
Comparison of model values with experimental results for the solution temperature.

Experimental data from the studies of Englart [17] and Englart and Rajski [28] were used to validate the MEC and MD models. The calculations were carried out using the same operating conditions as those given in the experiments. A comparison of the results obtained from the model with the experimental results for the evaporative cooling process is shown in Figure 4 and Figure 5. Figure 6 and Figure 7 show a comparison of the results obtained from the model with the experimental results for the air dehumidification. A good level of agreement between the model and experimental results was found. The relative error did not exceed 3% for all the analyzed parameters, including the air temperature, humidity ratio, water temperature, and solution temperature.

### 2.3. Performance Indices

The energy efficiency of the analyzed system can be described based on the coefficient of performance (COP). The COP system can be defined as the ratio of the supply cooling capacity to the total electrical power consumption of the fans and pumps:

(41) $$COP=\frac{{\dot{Q}}_{\sup}}{{\dot{P}}_{tot}}$$

The cooling capacity of the supply air can be determined as:

(42) $${\dot{Q}}_{\sup}={\dot{m}}_{{}_{\sup}}c_{pa}\left(t_{a1}-t_{a3}\right)$$

where:

(43) $${\dot{m}}_{{}_{\sup}}={\dot{m}}_{{}_{a,EC}}+{\dot{m}}_{{}_{a,D}}$$

The total electrical power consumption of the fans and pumps in the system can be determined by Equation (44):

(44) $${\dot{P}}_{{}_{tot}}={\dot{P}}_{{}_{F}}+{\dot{P}}_{{{}_{P}}_{,w}}+{\dot{P}}_{{}_{P,s}}$$

Hence, the electrical power required for air transfer can be represented by the following formula:

(45) $${\dot{P}}_{{}_{F}}=\frac{{\dot{m}}_{{}_{a}}\Delta p_{a}}{\rho_{a}\eta }$$

The detailed equations for the calculation of the air pressure difference between the inlet and outlet are described in [14,17].

The electricity power consumption for the water and solution pumping system can be calculated using the following equations, respectively:

(46) $${\dot{P}}_{{}_{P,w}}=\frac{{\dot{m}}_{{}_{w}}\Delta p_{P,w}}{\rho_{w}\eta },{\dot{P}}_{{}_{P,s}}=\frac{{\dot{m}}_{{}_{s}}\Delta p_{P,s}}{\rho_{s}\eta }$$

## 3. Results and Discussion

**Figure 8:**
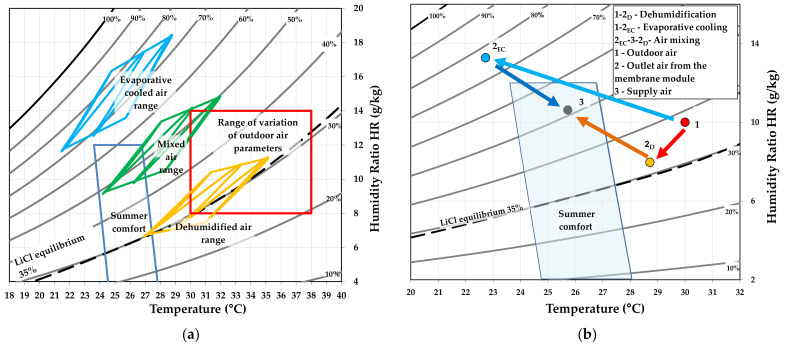
Psychrometric chart for the evaporative cooling, dehumidification, and air mixing processes: (**a**) for the whole analyzed range of the outdoor air; (**b**) for one initial point.

The proposed model allows us to perform an analysis of the performance of the system on the supply side of the fresh air, as well as on the regeneration side of the solution. The membrane-based fresh air processing system was evaluated in terms of the parameters summarized in Table 6. The range of the variation in the outdoor air temperature was from 30 °C to 38 °C and the humidity ratio was from 8 g/kg to 14 g/kg, as shown in Figure 8a.

Figure 8a also shows the ranges of the air parameters obtained for the dehumidification, evaporative cooling, and air mixing processes. Figure 8b shows the transformation of the air in a membrane-based fresh air processing system with the initial parameters t_a1_ = 30 °C and **ω**_a1_ = 10 g/kg (number 1 in Figure 8b). Points 2_D_ and 2_EC_ indicate the air parameters after dehumidification and evaporative cooling, respectively. Point 3 is the supply air parameters obtained by mixing the two air streams. As can be seen, after mixing, the supply air flow partially moves within the summer comfort zone (Figure 8a).

**Figure 9:**
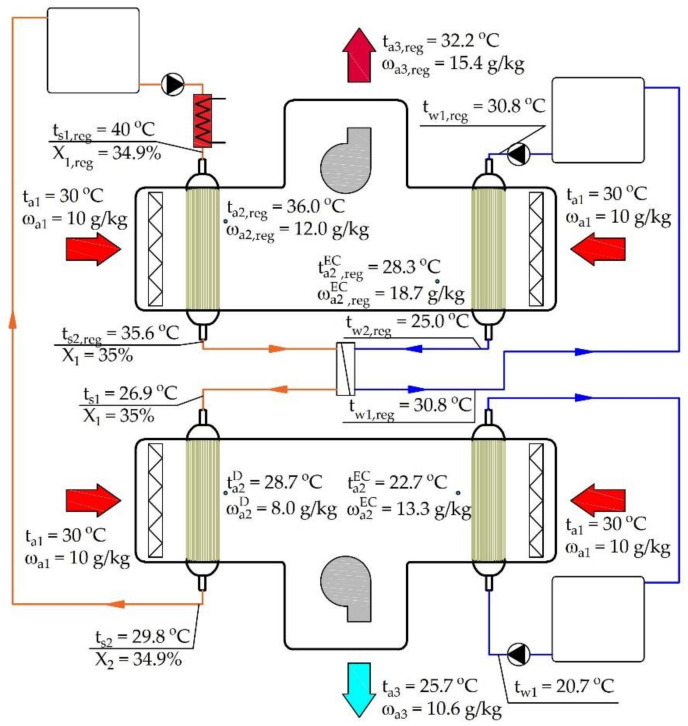
Examples of air and liquid parameters.

Figure 9 shows the modeling results for outdoor air of t_a1_ = 30 °C and ω_a1_ = 10 g/kg (point 1 of Figure 8b) and a solution regeneration temperature of t_s1,reg_ = 40 °C.

The use of evaporative cooling over the analyzed range of outdoor air temperatures and humidity alone resulted in a satisfactory drop in the air temperature, with a corresponding, but not acceptable, increase in the moisture content (Figure 8a). Carrying out the dehumidification process alone resulted in a slight decrease in the air temperature (at low outdoor air humidity ratios), as well as an increase in the air temperature (at high humidity ratios). Mixing the dehumidified and evaporatively cooled air streams allowed us to decrease the air temperature without a significant increase in the humidity ratio (Figure 8a).

### 3.1. Effects of the Inlet Air Temperature and Humidity Ratio

The differences in the temperature and humidity ratio for dehumidification, evaporative cooling, and stream mixing were defined, respectively, as:

(47) $$\Delta T_{a}=\left(t_{a1}-t_{a2}\right)$$

(48) $$\Delta \omega_{a}=\left(\omega_{a2}-\omega_{a1}\right)$$

**Figure 10:**
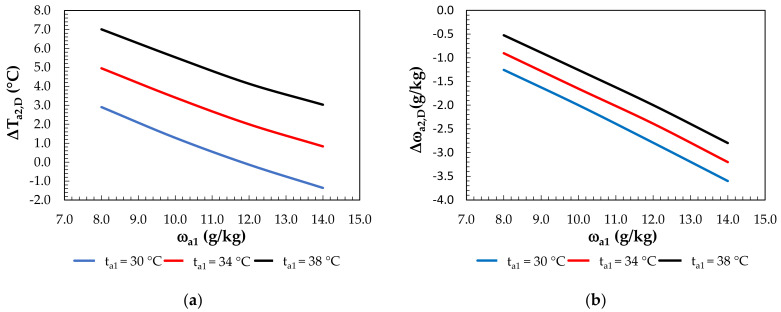
Effect of the inlet air humidity ratio for constant inlet air temperatures under air dehumidification: (**a**) on air temperature differences; (**b**) on the change in the humidity ratio.

Figure 10 shows the change in the air parameters after dehumidification as a function of the humidity ratio and outside air temperature. Negative values indicate an increase and positive values indicate a decrease in the dehumidified air temperature (Figure 10a). In Figure 10b, negative values indicate air dehumidification.

The change in the humidity ratio ranged from 0.5 g/kg (for t_a1_ = 38 °C and at ω_a1_ = 8 g/kg) to 3.6 g/kg (for t_a1_ = 30 °C and at ω_a1_ = 14 g/kg). In the air dehumidification process, the highest temperature drop occurred at the lowest humidity ratio (ω_a1_ = 8 g/kg) and highest temperature (t_a1_ = 38 °C). This was due to the fact that the lowest solution temperature was obtained in the regeneration process for this humidity ratio. The higher the humidity ratio is, the higher the solution regeneration temperature required must be.

**Figure 11:**
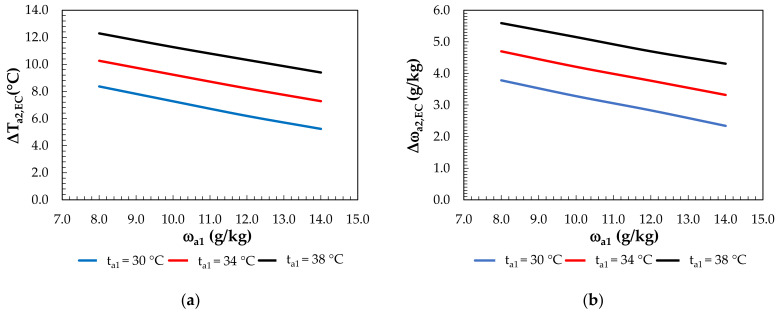
Effect of the inlet air humidity ratio for constant inlet air temperatures under air evaporative cooling: (**a**) on air temperature differences; (**b**) on the change in the humidity ratio.

For the evaporative cooling (Figure 11), the temperature drop ranged from 5.2°C (at t_a1_ = 30 °C, ω_a1_ = 14 g/kg) to 12.3 (at t_a1_ = 38 °C, ω_a1_ = 8 g/kg), while the increase in the humidity ratio ranged from 2.3 g/kg (at t_a1_ = 30°C, ω_a1_ = 14 g/kg) to 5.6 g/kg (at t_a1_ = 38 °C, ω_a1_ = 8 g/kg).

**Figure 12:**
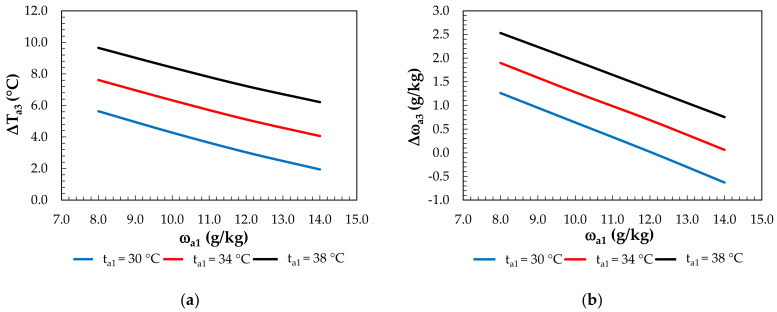
Effect of the inlet air humidity ratio for constant inlet air temperatures under mixing: (**a**) on air temperature differences; (**b**) on the change in the humidity ratio.

Mixing the streams (Figure 12) of dehumidified and evaporatively cooled air slightly decreased the temperature drop compared to evaporative cooling. The temperature drop ranged from 1.9 °C to 9.7 °C, with a corresponding decrease in the air humidity from 5.6 g/kg to 2.5 g/kg (at t_a1_ = 38°C, ω_a1_ = 8 g/kg). For t_a1_ = 30 °C and ω_a1_ = 14 g/kg, there was slight air dehumidification of Δω = −0.6 g/kg.

### 3.2. Analysis of the Regeneration System’s Operation

**Figure 13:**
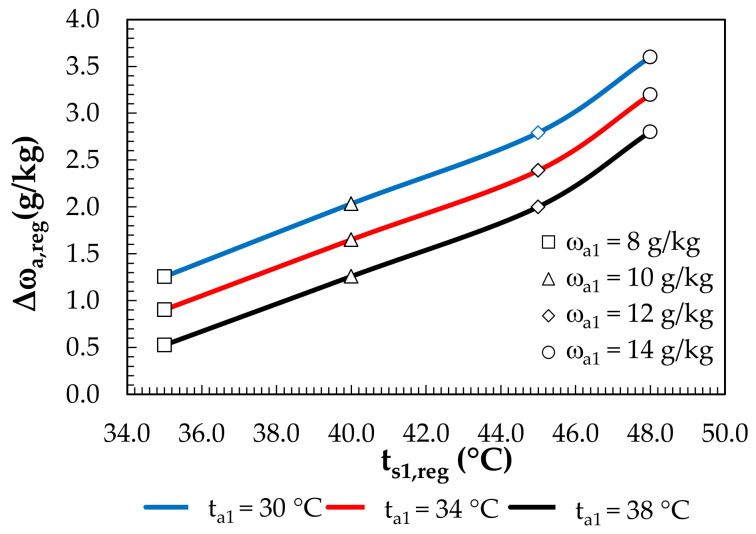
Effects of the solution temperature at the regenerator inlet and regeneration air parameters on the change in the humidity ratio.

The inlet solution temperature range for the regeneration was from 35 °C at a regeneration air humidity ratio of 8 g/kg to 48 °C at 14 g/kg. In the regeneration air, there was an increase in the humidity ratio from 0.5 g/kg (for t_a1_ = 38 °C, ω_a1_ = 8 g/kg) to 3.6 g/kg (for t_a1_ = 30 °C, ω_a1_ = 14 g/kg), which indicates the effective regeneration of the solution (Figure 13).

**Figure 14:**
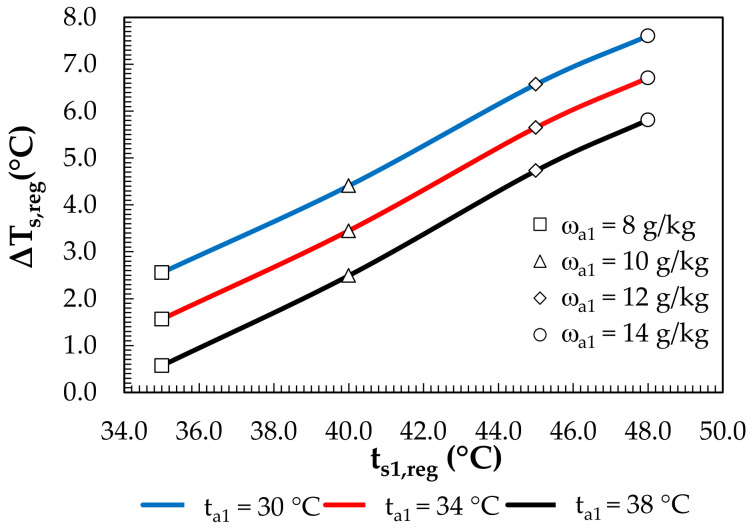
Effects of the solution temperature at the regenerator inlet and regeneration air parameters on the solution temperature drop.

**Figure 15:**
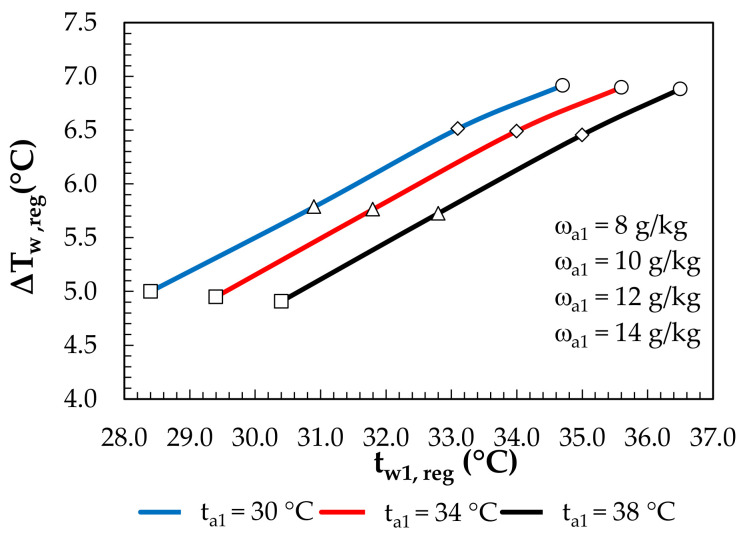
Water temperature drop in the MEC depending on the outdoor air parameters and inlet water temperature.

The drop in the solution temperature during regeneration is shown in Figure 14. It ranged from 0.6 °C to 7.6 °C. A further decrease in the solution temperature was obtained in the PHE, whereas the preheated water in the PHE was cooled in MEC. The drop in the water temperature obtained for the MEC is shown in Figure 15. The drop in the water temperature ranged from 4.9 °C to 6.9 °C and was enough to supply the PHE.

### 3.3. Heat and Cooling Capacity of the Regeneration and COP of the System

**Figure 16:**
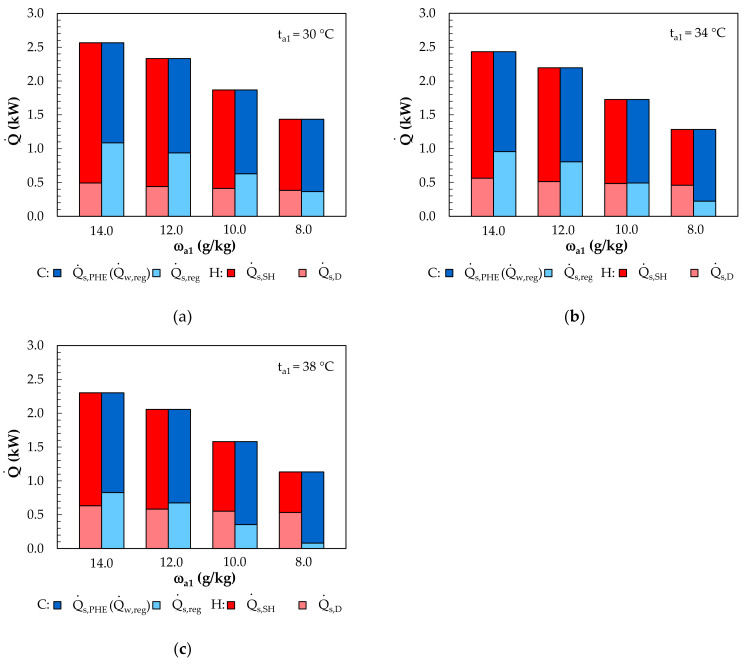
Effect of the inlet air humidity ratio on the heat and cooling capacity of the solution side (C–cooling, H–heating): (**a**) for an inlet air temperature of 30 °C; (**b**) for an inlet air temperature of 34 °C; (**c**) for an inlet air temperature of 38 °C.

The heat and cooling capacity demands of the solution loop are shown in Figure 16. The system analyzed assumes that the heating of the LiCl solution is achieved with solar collectors. The heat capacity required for heating the solution from the solar system, ${\dot{Q}}_{s,\mathrm{SH}}$, depended on the outdoor air parameters and ranged from 0.6 kW (t_a1_ = 38, ω_a1_ = 8 g/kg) to 2.07 kW (t_a1_ = 30, ω_a1_ = 14 g/kg). The higher the humidity ratio in the regeneration air is, the higher the temperature that the solution is heated to before regeneration must be. This is due to the need to ensure an adequate difference in the moisture content between the regeneration air and the point of the regeneration potential on the LiCl equilibrium curve. In addition, the appropriate regeneration of the solution must allow for the following condition to be achieved: Δω_a,reg_ = −Δω_a2,D_.

The cooling of the solution was carried out in an external system. The highest cooling capacity occurred at ω_a1_ = 14 g/kg and was in the range of ${\dot{Q}}_{w,\mathrm{reg}}$ = 1.47–1.48 kW, and the lowest, at ω_a1_ = 8 g/kg, was in the range of ${\dot{Q}}_{w,\mathrm{reg}}$ = 1.05–1.07 kW. A higher solution temperature with a higher moisture content resulted in a higher water temperature at the PHE outlet and, thus, a higher inlet temperature for the MEC.

**Figure 17:**
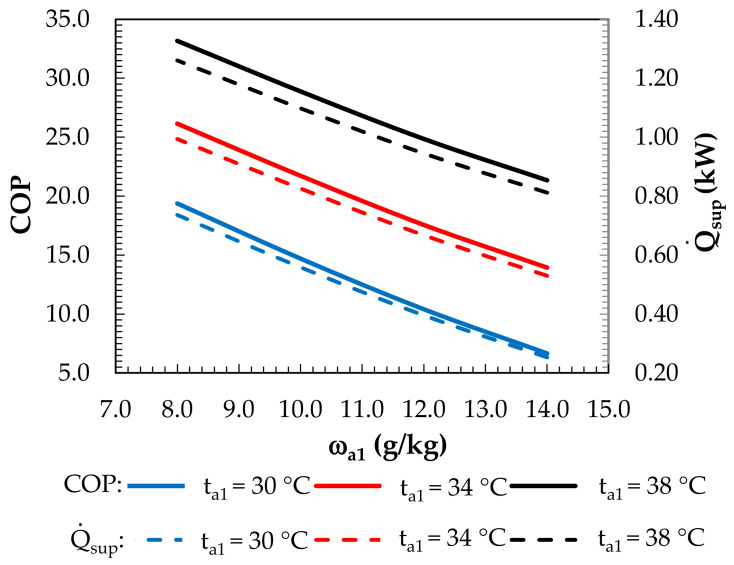
Effect of the inlet air humidity ratio for constant inlet air temperatures on the COP coefficient and supply cooling capacity.

The effects of the outdoor air parameters on the COP of the system and the cooling capacity of the supply air are shown in Figure 17. The highest COP and ${\dot{Q}}_{\sup}\;$ values were obtained at the lowest outdoor air humidity ratio and at a particular outdoor air temperature. In such conditions, the highest drop in the air temperature in the MEC was obtained, and the inlet temperature of the solution for the dehumidifier was the lowest, resulting in the lowest air mixture temperatures and, thus, the highest ${\dot{Q}}_{\sup}\;$ values.

## 4. Conclusions

This paper presented a membrane-based fresh air processing system. A complete unit for the cooling of the outdoor air and regeneration of the desiccant solution was described. To determine the performance of the novel approach, the operation of the system was modeled. The proposed mathematical model was validated using literature data. A system with a supply air flow rate of 460 kg/h was analyzed. The parallel combination of membrane modules enabled simultaneous evaporative cooling and outdoor air dehumidification. The system was tested under the following outdoor air parameters: a temperature ranging from 30 °C to 38 °C and humidity ratio ranging from 8 g/kg to 14 g/kg. The main conclusions of the research are as follows:

- The proposed model showed good agreement with the experimental data. The relative error did not exceed 3%;
- Using the system, an air temperature difference of up to 9.7 °C was achieved, with a slight increase in the humidity ratio;
- The adopted concept of the membrane-based fresh air processing system allowed us to obtain supply air parameters in or close to the summer comfort range;
- The analyzed system was characterized by a high COP of up to 33.2.

The main limitations of the results provided here relate to the climatic analysis, which creates room for further development. Thus, this study creates several areas for future research, including:

- A feasibility study of the investigated system in different climatic conditions;
- An optimization study of the membrane module geometries and processed air mixing;
- An energy, environmental, and economic analysis (3E).

## Figures and Tables

**Table 1:** Geometric parameters of the single hollow-fiber membrane module.

Parameters	Symbol	Unit	Value
Module length	L	m	0.4
Module width	W	m	0.1
Number of fibers	n_f_	-	744
Membrane inner diameter	d_i_	mm	1.67
Membrane outer diameter	d_o_	mm	2.71
Membrane thickness	δ	mm	0.52
Mean pore size	d_p_	μm	0.43
Maximum pore size	d_pmax_	μm	0.55
Effective area	A_tot_	m^2^	2.53
Packing density	A_v_	m^2^ m^−3^	0.4
Packing fraction	φ	-	0.1
Porosity	ε_p_	-	744
Tortuosity	τ	-	1.67

**Table 2:** Mathematical model equations for membrane evaporative cooler (MEC).

Energy Balance/Parameter	Equation	No.
Conservation of energy in the air stream	${\dot{m}}_{{}_{a}}c_{pa}dt_{a}=-H_{ol}\left(t_{a}-t_{w}\right)\frac{A_{tot}}{W}dx$	(1)
Conservation of moisture in the air stream	${\dot{m}}_{{}_{a}}d\omega_{a}=K_{ol}\rho \left(\omega_{w}-\omega_{a}\right)\frac{A_{tot}}{W}dx$	(2)
Conservation of energy in the water stream	${\dot{m}}_{{}_{w}}c_{pw}dt_{w}=H_{ol}\left(t_{a}-t_{w}\right)\frac{A_{tot}}{L}dy-h_{v}K_{ol}\rho_{a}\left(\omega_{w}-\omega_{a}\right)\frac{A_{tot}}{L}dy$	(3)
Humidity ratio of the saturated humid air [21,22]	$\omega_{w}=\frac{10^{6}}{e^{5294/\left(t_{w}+273.15\right)}}$	(4)

**Table 3:** Mathematical model equations for membrane dehumidifier (MD)/membrane regenerator (MR).

Energy Balance/Parameter	Equation	No.
Conservation of energy in the air stream	${\dot{m}}_{{}_{a}}c_{pa}dt_{a}=-H_{ol}\left(t_{a}-t_{s}\right)\frac{A_{tot}}{W}dx$	(5)
Conservation of moisture in the air stream	${\dot{m}}_{{}_{a}}d\omega_{a}=K_{ol}\rho \left(\omega_{s}-\omega_{a}\right)\frac{A_{tot}}{W}dx$	(6)
Conservation of energy in the solution stream	${\dot{m}}_{{}_{s}}c_{ps}dt_{s}=H_{ol}\left(t_{a}-t_{s}\right)\frac{A_{tot}}{L}dy-h_{abs}K_{ol}\rho_{a}\left(\omega_{s}-\omega_{a}\right)\frac{A_{tot}}{L}dy$	(7)
Conservation of moisture in the solution stream	$\left({\dot{m}}_{{}_{s}}+{\dot{m}}_{{}_{a}}d\omega_{a}\right)dX=K_{ol}\rho_{a}\left(\omega_{s}-\omega_{a}\right)X_{1}\frac{A_{tot}}{L}dy$	(8)
Humidity ratio of the saturated humid air [21]	$\omega_{s}=\frac{\phi 10^{6}}{e^{5294/\left(t_{s}+273.15\right)}}$	(9)
Absorption heat [23]	$h_{abs}=h_{v}+\Delta h_{mix}$	(10)

**Table 4:** Details of parameters for determining the individual resistance.

Parameter	Equation	No.
Heat transfer coefficient on the solution side	$h_{l}=\frac{Nu_{s}\lambda_{s}}{d_{i}}$	(14)
Nusselt number for the flow in hollow-fiber membrane [21]	Nus=3.658+0.085RePrdi/L1+0.047RePrdi/L0.67μfμW0.14	(15)
Equivalent moisture transfer coefficient in the solution [25]	$k_{le}=k_{l}K_{p}\frac{\rho_{s}}{\rho_{a}}$	(16)
Mass transfer coefficient on the solution side [26]	$k_{l}=1.62{\left(\frac{vD_{s}^{2}}{Ld_{i}}\right)}^{0.33}$	(17)
Convective mass transfer coefficient on the air side	$k_{a}=\frac{Sh_{a}D_{f}}{d_{o}}$	(18)
Sherwood number for the shell side of the hollow-fiber membrane [27]	$Sh_{a}=5.85(1-\varphi )\left(\frac{d_{h}}{L}\right)Re^{0.6}Sc^{0.33}$	(19)
Convective heat transfer coefficient on the air side	$h_{a}=\frac{Nu_{a}\lambda_{a}}{d_{o}}$	(20)
Nusselt number for the shell side of the hollow-fiber membrane [17]	$Nu_{a}=Sh_{a}{\left(\frac{Pr}{Sc}\right)}^{1/3}$	(21)
Membrane thermal conductivity	$\lambda_{m}=\varepsilon_{p}\lambda_{a}+\left(1-\varepsilon_{p}\right)\lambda_{so}$	(22)
Effective moisture diffusivity in the membrane	$D_{vm}=\frac{\varepsilon_{p}D}{\tau }$	(23)

**Table 5:** Heat and cooling balance of the LiCl side.

Heat and Cooling Balance	Equation	No.
The total heating capacity of LiCl heating	${\dot{Q}}_{Hs,tot}={\dot{Q}}_{s,D}+{\dot{Q}}_{s,SH}$	(28)
The heating capacity of LiCl in the dehumidifier	${\dot{Q}}_{s,D}={\dot{m}}_{{}_{s}}c_{ps}\left(t_{s2}-t_{s1}\right)$	(29)
The heating capacity of LiCl in the solar heat exchanger	${\dot{Q}}_{s,SH}={\dot{m}}_{{}_{s}}c_{ps}\left(t_{s1,reg}-t_{s2}\right)$	(30)
The total cooling capacity of LiCl cooling	${\dot{Q}}_{Cs,tot}={\dot{Q}}_{s,reg}+{\dot{Q}}_{s,PHE}$	(31)
The cooling capacity of LiCl in the regenerator	${\dot{Q}}_{s,reg}={\dot{m}}_{{}_{s}}c_{ps}\left(t_{s1,reg}-t_{s2,reg}\right)$	(32)
The cooling capacity of LiCl cooling in PHE	${\dot{Q}}_{s,PHE}={\dot{m}}_{{}_{s}}c_{ps}\left(t_{s2,reg}-t_{s1}\right)$	(33)
PHE heat balance	${\dot{Q}}_{s,PHE}={\dot{Q}}_{w,reg}$	(34)
The cooling capacity of water cooling in MEC	${\dot{Q}}_{w,reg}={\dot{m}}_{{}_{w}}c_{pw}\left(t_{w1,reg}-t_{w2,reg}\right)$	(35)

**Table 6:** Operating parameters for the membrane-based fresh air processing system.

Parameters	Symbol	Unit	Value/Range
Dehumidified air flow	${\dot{m}}_{a,D}$	kg/h	230
Evaporative cooled air flow	${\dot{m}}_{a,\mathrm{EC}}$	kg/h	230
Supply air flow	${\dot{m}}_{\sup}$	kg/h	460
Solution flow rate	${\dot{m}}_{s}$	kg/h	184
Water flow rate	${\dot{m}}_{w}$	kg/h	184
The ratio of air mass flow to liquid mass flow	m^*^	-	1.25
Outdoor air temperature	t_a1_	°C	30–38
Outdoor air humidity ratio	**ω**_a1_	g/kg	8–14
The concentration of the solution at the MD inlet	X_1_	%	35
The temperature of the solution at the membrane regenerator inlet	t_s1,reg_	°C	35–48
The total area of membrane modules	A_tot_	m^2^	25.3
Number of transfer units of PHE	NTU	-	2.83

## Data Availability

Data is contained within the article.

## Nomenclature

A	area, m^2^
c_p_	specific heat, J kg^−1^ K^−1^
D	diffusivity, m^2^ s^−1^
d	diameter, m
d_p_	mean pore diameter, m
D_vm_	effective moisture diffusivity, m^2^ s^−1^
H_ol_	overall heat transfer, W m^−2^ K^−1^
h	heat transfer coefficient, W m^−2^ K^−1^
h_abs_	absorption heat, J kg^−1^
h_mix_	enthalpy of mixing, J kg^−1^
h_v_	heat of evaporation of water, J kg^−1^
K_ol_	overall mass transfer, m s^−1^
K_p_	partition coefficient of a solution
k	mass transfer coefficient, m s^−1^
L	length of hollow fiber, m
$\dot{m}$	mass flow rate, kg s^−1^
m*	ratio of air mass flow to solution mass flow
NTU	number of transfer units
Nu	Nusselt number
$\dot{P}$	electric power demand, W
p	pressure, Pa
Pr	Prandtl number
R	resistance
$\dot{Q}$	heating capacity, cooling capacity, W
Re	Reynolds number
Sc	Schmidt number
Sh	Sherwood number
t	temperature, °C
W	width of membrane module, m
x, y	axial coordinates, m
**Greek Letters**	
ε	effectiveness
ε_p_	porosity
φ	packing fraction
ϕ	concentration of water
ρ	density, kg m^−3^
τ	tortuosity
η	device efficiency
λ	heat conductivity, W m^−1^ K^−1^
ω	humidity ratio, kg kg^−1^
μ	dynamic viscosity, Pa s
δ	membrane thickness, m
**Subscripts**	
1	inlet
2	outlet
3	mixed
a	air
EC	evaporative cooling
exp	experimental
D	dehumidification
f	fluid
F	fan
h	hydraulic
i	inner
l	liquid
ln	logarithmic
m	membrane
mod	model
o	outer
P	pump
reg	regeneration
s	solution
so	solid
sup	supply
tot	total
w	water
W	wall

## References

[1] Guyot G., Sherman M.H., Walker I.S. (2018). Smart Ventilation Energy and Indoor Air Quality Performance in Residential Buildings: A Review. Energy Build..

[2] Al-Rawi M., Ikutegbe C.A., Auckaili A., Farid M.M. (2021). Sustainable Technologies to Improve Indoor Air Quality in a Residential House—A Case Study in Waikato, New Zealand. Energy Build..

[3] Niu R.P., Chen X., Liu H. (2022). Analysis of the Impact of a Fresh Air System on the Indoor Environment in Office Buildings. Sustain. Cities Soc..

[4] González-Torres M., Pérez-Lombard L., Coronel J.F., Maestre I.R., Yan D. (2022). A Review on Buildings Energy Information: Trends, End-Uses, Fuels and Drivers. Energy Rep..

[5] Derbez M., Berthineau B., Cochet V., Lethrosne M., Pignon C., Riberon J., Kirchner S. (2014). Indoor Air Quality and Comfort in Seven Newly Built, Energy-Efficient Houses in France. Build. Environ..

[6] Zhao L., Liu J., Ren J. (2018). Impact of Various Ventilation Modes on IAQ and Energy Consumption in Chinese Dwellings: First Long-Term Monitoring Study in Tianjin, China. Build. Environ..

[7] Huang K., Sun W., Feng G., Wang J., Song J. (2020). Indoor Air Quality Analysis of 8 Mechanically Ventilated Residential Buildings in Northeast China Based on Long-Term Monitoring. Sustain. Cities Soc..

[8] Yan H., Chen Y., Min Y., Pan Y. (2022). An Adaptive Controller Based Dynamic Simulation of Household Air-Conditioner with Indirect Evaporative Cooler as Dedicated Outdoor Air System. Energy Build..

[9] Bai H., Zhu J., Chen Z., Chu J. (2018). State-of-Art in Modelling Methods of Membrane-Based Liquid Desiccant Heat and Mass Exchanger: A Comprehensive Review. Int. J. Heat Mass Transf..

[10] Gurubalan A., Maiya M.P., Geoghegan P.J. (2019). A Comprehensive Review of Liquid Desiccant Air Conditioning System. Appl. Energy.

[11] Fu H.-X., Liu X.-H. (2017). Review of the Impact of Liquid Desiccant Dehumidification on Indoor Air Quality. Build. Environ..

[12] Liu X., Qu M., Liu X., Wang L. (2019). Membrane-Based Liquid Desiccant Air Dehumidification: A Comprehensive Review on Materials, Components, Systems and Performances. Renew. Sustain. Energy Rev..

[13] Peng D., Cao Z. (2021). Modeling and Performance Analysis of a Hybrid-Connected Two-Stage Liquid Dehumidification Fresh Air System Based on CaCl_2_/LiCl Double Solution. Appl. Therm. Eng..

[14] Englart S., Rajski K. (2022). A Novel Membrane Liquid Desiccant System for Air Humidity Control. Build. Environ..

[15] Zhang N., Chen X., Su Y., Zheng H., Ramadan O., Zhang X., Chen H., Riffat S. (2019). Numerical Investigations and Performance Comparisons of a Novel Cross-Flow Hollow Fiber Integrated Liquid Desiccant Dehumidification System. Energy.

[16] Li N., Zhong T., Zhou L., Huang S., Zeng S., Liang C. (2022). Experimental Investigations on the Performance of a Hollow Fiber Membrane Evaporative Cooler (HFMEC) in Hot–Dry Regions. Membranes.

[17] Englart S. (2020). Use of a Membrane Module for Semi-Direct Air Evaporative Cooling. Indoor Built Environ..

[18] Cui X., Yan W., Liu Y., Zhao M., Jin L. (2020). Performance Analysis of a Hollow Fiber Membrane-Based Heat and Mass Exchanger for Evaporative Cooling. Appl. Energy.

[19] Jradi M., Riffat S. (2016). Testing and Performance Analysis of a Hollow Fiber-Based Core for Evaporative Cooling and Liquid Desiccant Dehumidification. Int. J. Green Energy.

[20] Abdollahi F., Hashemifard S.A., Khosravi A., Matsuura T. (2021). Heat and Mass Transfer Modeling of an Energy Efficient Hybrid Membrane-Based Air Conditioning System for Humid Climates. J. Membr. Sci..

[21] Yang B., Yuan W., Gao F., Guo B. (2015). A Review of Membrane-Based Air Dehumidification. Indoor Built Environ..

[22] Zhang L.-Z., Huang S.-M. (2011). Coupled Heat and Mass Transfer in a Counter Flow Hollow Fiber Membrane Module for Air Humidification. Int. J. Heat Mass Transf..

[23] Conde M.R. (2004). Properties of Aqueous Solutions of Lithium and Calcium Chlorides: Formulations for Use in Air Conditioning Equipment Design. Int. J. Therm. Sci..

[24] Johnson D.W., Yavuzturk C., Pruis J. (2003). Analysis of Heat and Mass Transfer Phenomena in Hollow Fiber Membranes Used for Evaporative Cooling. J. Membr. Sci..

[25] Zhang L.-Z. (2013). Heat and Mass Transfer Across a Hollow Fiber Membrane Bundle. Conjugate Heat and Mass Transfer in Heat Mass Exchanger Ducts.

[26] Mahmud H., Kumar A., Narbaitz R.M., Matsuura T. (2000). A Study of Mass Transfer in the Membrane Air-Stripping Process Using Microporous Polyproplylene Hollow Fibers. J. Membr. Sci..

[27] Prasad R., Sirkar K.K. (1988). Dispersion-Free Solvent Extraction with Microporous Hollow-Fiber Modules. AIChE J..

[28] Englart S., Rajski K. (2021). Performance Investigation of a Hollow Fiber Membrane-Based Desiccant Liquid Air Dehumidification System. Energies.

